# Disseminated cutaneous infection

**DOI:** 10.11604/pamj.2016.25.259.10799

**Published:** 2016-12-29

**Authors:** Juan Carlos Cataño, Veronica Posada

**Affiliations:** 1 Infectious Diseases Section, Internal Medicine Department, University of Antioquia School of Medicine, Medellín, Colombia; 2 Infectious Diseases Section, CES Clinic, Medellín, Colombia

**Keywords:** Botryomycosis, infection, HIV

## Image in medicine

A 65 year-old man with no remarkable medical history, presented to the emergency room with 5 months of intermittent growth of skin nodules that last about 2 months and heal spontaneously, associated with high spiking fevers, dyspnea, dry cough and 8 Kg weight loss. Physical examination demonstrated noteworthy cachexia, oral thrush, bibasal crackles and multiple non-tender subcutaneous nodules in the trunk and extremities. The nodules were erithemato-violaceous, necrotic and ulcerated, with copious discharge of purulent material (A, B). Computed tomography scans of the neck, chest and abdomen were normal except for patchy ground-glass infiltrates in the lungs. Laboratory data showed anemia 8.7 mg/dL, leukopenia, a positive human immunodeficiency virus (HIV) serology, CD4 count of 27 cells/uL, and Silver-methenamine staining of bronchoalveolar lavage with *pneumocystis jirovecii* cysts. Biopsy of the lesions was performed and optical microscopy examination with hematoxylin-eosin staining demonstrated epidermal acanthosis and dermal fibrosis with suppurative and chronic inflammation, eosinophilic, amorphous granules or grains containing clusters of nonfilamentous bacteria arranged in lobules (C), but no signs of malignant neoplasia or other microorganisms were informed, and bacterial cultures grew *Staphylococcus aureus*. According to these findings, the diagnosis of cutaneous botryomycosis was made, and intravenous antimicrobial therapy with Trimethoprim-Sulfamethoxazole was initiated, showing a dramatic improvement of the lesions after only 8 days of treatment (D), then the patient was discharged with oral antibiotic therapy with Trimethoprim-Sulfamethoxazole and antiretroviral medication.

**Figure 1:**
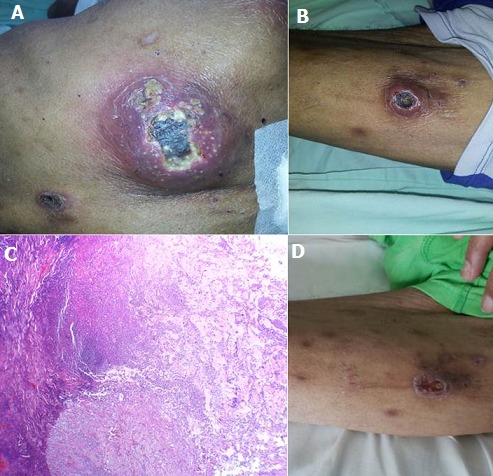
A) erithemato-violaceous nodules, necrotic and ulcerated; B) with copious discharge of purulent material; C) hematoxylin-eosin staining demonstrated epidermal acanthosis and dermal fibrosis with suppurative and chronic inflammation, eosinophilic, amorphous granules or grains containing clusters of nonfilamentous bacteria arranged in lobules; D) clinical improvement of the lesions after 8 days of treatment

